# Inflammatory myofibroblastic tumor of the temporal bone presenting with pulsatile tinnitus: a case report

**DOI:** 10.1186/1752-1947-7-157

**Published:** 2013-06-20

**Authors:** Xiaoli Zhou, Tingting Liu, Zhibin Chen, Zhihong Zhang, Guangqian Xing

**Affiliations:** 1 Department of Otolaryngology, First Affiliated Hospital of Nanjing Medical University, 300 Guangzhou Road, Nanjing, 210029, China; 2 Department of Pathology, First Affiliated Hospital of Nanjing Medical University, Nanjing, 210029, China

**Keywords:** Inflammatory Myofibroblastic Tumor, Middle Ear, Pulsatile Tinnitus, Temporal Bone

## Abstract

**Introduction:**

Inflammatory myofibroblastic tumor of the temporal bone is an unusual but distinct disease entity. The most common presenting symptoms are otalgia, otorrhea, hearing loss, facial palsy, and vertigo. We describe here what we believe to be the first reported case of a patient presenting with persistent pulsatile tinnitus. The clinical features, radiological and histopathologic findings, and treatment outcomes of the patient are presented.

**Case presentation:**

A 59-year-old woman of Chinese Han origin presented with complaints of left-sided pulsatile tinnitus and progressive hearing loss for several years. Clinical evaluations revealed a reddish mass behind the intact tympanic membrane, and a moderately severe conductive hearing loss in the left ear. The computed tomographic imaging of the temporal bone demonstrated a slightly ill-defined left middle ear soft tissue mass involving the posterior portion of the mesotympanum and epitympanum, and the mastoid antrum. The patient underwent surgical excision of the lesion which subsequently resolved her symptoms. Postoperative pathology was consistent with an inflammatory myofibroblastic tumor.

**Conclusions:**

An inflammatory myofibroblastic tumor of the temporal bone can present clinically with pulsatile tinnitus and masquerade as venous hum or vascular tumors of the middle ear; therefore, it should be included in the differential diagnosis of pulsatile tinnitus.

## Introduction

Inflammatory myofibroblastic tumors (IMFT), also known as inflammatory pseudotumors, include a diverse group of lesions characterized by inflammatory cell infiltration and variable fibrotic responses [1]. It is an unusual histologically benign yet clinically invasive mass lesion of unknown etiology that most frequently involves the lung, but it has been described in almost any location, in both genders, at all ages [2,3]. IMFT of the head and neck are uncommon, accounting for fewer than 5% of all extrapulmonary cases. In the head and neck, the most common location is the orbit, followed by the meninges, paranasal sinuses, infratemporal fossa, and soft tissues. The temporal bone, skull base, and facial nerve are very rarely involved [3,4]. A recent review of the literature by Ajibade *et al*. identified only 35 such cases [4]. However, IMFT of the temporal bone appears to have a more aggressive and unpredictable course than those in other body sites. IMFT occurring in the middle ear and mastoid may erode into the surrounding dura, sigmoid sinus, tentorium, and even brain parenchyma [3,5-8]. Intratemporal extensions to the otic capsule, facial nerve, petrous apex, and internal auditory canal are also common [3,8-10]. This makes the clinical manifestations of this rare disease entity more variable, and the diagnosis and the treatment more challenging than IMFT of the lung.

In this article we describe what we believe is the first reported case with an IMFT in a 59-year-old woman presenting with persistent pulsatile tinnitus. The clinical features, radiological and histopathologic findings, and treatment outcomes are presented.

## Case presentation

A 59-year-old woman of Chinese Han origin presented with a complaint of a left pulsatile tinnitus. She had been extremely depressed for 10 years because this tinnitus was constant and prevented her from sleeping. She had visited the otological clinics at different hospitals several times. At each presentation, the tympanic membrane was carefully examined otoscopically and was found to be normal. A computed tomographic (CT) scan of the temporal bone was also performed 9 years prior to her presentation to our hospital with a negative finding. With the initial diagnosis of venous hum, she received no effective treatment.

**Figure 1:**
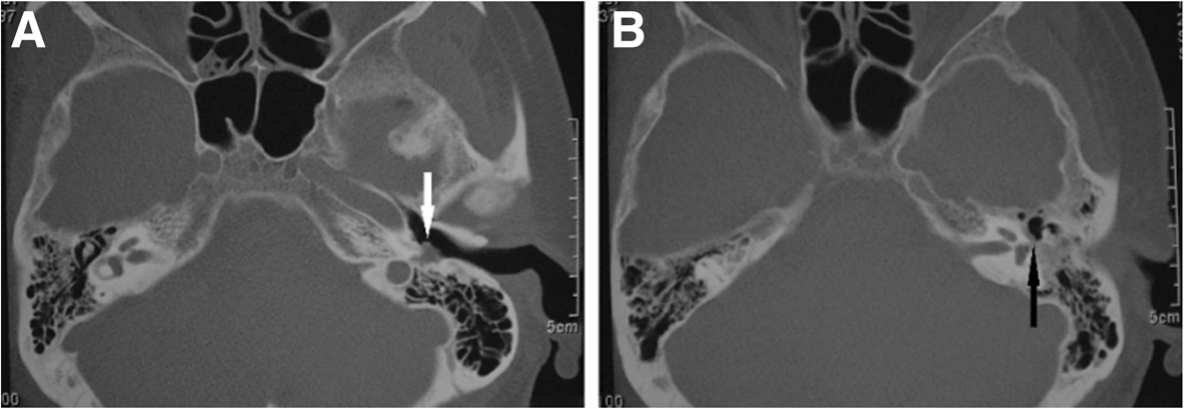
**Axial computed tomography of the temporal bone shows a slightly ill-defined left middle ear soft tissue mass involving (A) the posterior mesotympanum *(white arrow)*, and (B) the posterior epitympanum and mastoid antrum with erosion of the facial fallopian canal around the second genu *(black arrow).***

The patient was referred to us for further evaluation on January 17, 2007, due to the unbearable left tinnitus and a new awareness that her hearing had gradually deteriorated for 2 years. Her medical history was otherwise unremarkable except for hypertension, which was under treatment. On examination, the pulsatile tinnitus could be completely suppressed by digital pressure over the ipsilateral internal jugular vein or when the patient was asked to turn her head towards the side of tinnitus. Cranial, carotid and chest auscultation were negative. Other examinations including carotid ultrasonography, electrocardiography, thyroid function tests, and chest X-ray were normal. Careful otoscopic examination revealed a vague and reddish mass behind the superior-posterior portion of the intact left tympanic membrane. A pure tone audiogram showed a moderately severe conductive hearing loss in the left ear, especially at lower frequencies; middle ear impedance testing ascertained a flat tympanogram of the same ear. High resolution CT scans of the temporal bone showed a slightly ill-defined left middle ear soft tissue mass involving the posterior mesotympanum and epitympanum, and the mastoid antrum with erosive changes of the facial fallopian canal (Figure 1). The margin between the lesion and incus was vague. There was no erosion of the lateral semicircular canal.

With the suspicion of a neoplastic lesion, the patient underwent an exploration surgery through a postauricular approach. During this procedure, the lesion was noted to center in the fossa incudis which apparently encased the incus and eroded through the second genu of the fallopian canal, but the lateral semicircular canal and facial nerve were not involved. There was extension of the mass into the posterior mesotympanum. Intraoperative frozen sections were obtained and documented a chronic inflammation. The patient then underwent complete removal of the lesion followed by stage I ossicular reconstruction. The facial nerve was carefully decompressed meanwhile without incision of the neural sheath.

**Figure 2:**
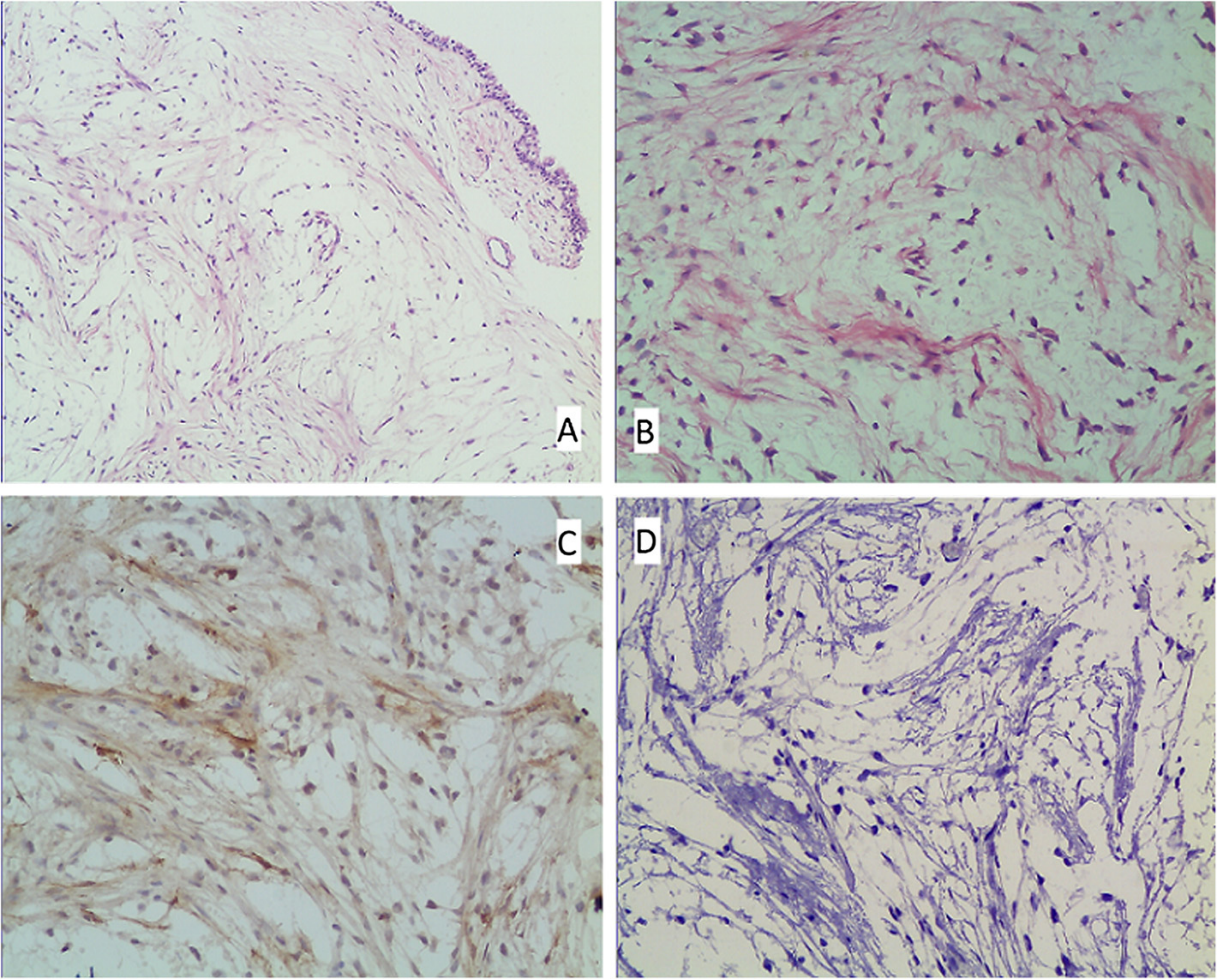
**Histopathologic findings.** Spindle-shaped cells (myofibroblasts and fibroblasts) loosely arranged in an edematous and mucous-degenerated stroma (hematoxylin and eosin **A**: × 100; **B**: × 200). Immunohistochemical stain shows a positive reaction of the spindle cells for smooth muscle actin (**C**: × 200), and negative reaction for activin receptor-like kinase 1 (**D**: × 200).

The histopathologic study of the mass showed proliferation of myofibroblasts and fibroblasts within an edematous and mucous-degenerated stroma, with scattered infiltration of inflammatory cells (Figure 2A-B); mitotic activity was not observed. On immunohistochemistry, the spindle cells stained with smooth muscle actin (Figure 2C) but were negative for activin receptor-like kinase 1 (Figure 2D), epithelial membrane antigen, desmin, ki-67 and S-100. These features are consistent with an IMFT.

The postoperative course was uneventful, and the patient’s pulsatile tinnitus disappeared immediately after surgery. She was discharged home at the third postoperative day. During a 5-year follow-up period, no signs of recurrence were noted.

## Discussion

The causes of pulsatile tinnitus are numerous. An IMFT of the temporal bone presenting with pulsatile tinnitus represents a diagnostic and therapeutic dilemma because of its rarity. Clinical history and physical examination are of the utmost importance in establishing the correct diagnosis. For patients with an otoscopically visible retrotympanic lesion, a CT scan of the temporal bone should be ordered to detect underlying causes, these include [11-14]: (1) vascular tumors of the middle ear, such as glomus tympanicus, glomus jugulare tumor, and hemangioma; (2) vascular abnormalities, such as high jugular bulb, an aberrant carotid artery or persistent stapedial artery; and (3) inflammatory or other neoplastic lesions, such as cholesterol granuloma, aural polyp, meningioma, and occasionally middle ear adenoma.

The patient we present here was a 59-year-old woman. She had a complaint of left-sided pulsatile tinnitus for 10 years, and recently a progressive hearing loss in the same ear for 2 years. Otoscopic examination revealed a reddish middle ear mass behind her left tympanic membrane. CT scans of the temporal bone demonstrated a soft tissue mass around the incus with extension into the posterior portion of the mesotympanum and epitympanum. For that reason, the clinical suggestion was a possible neoplastic or inflammatory lesion, especially a vascular tumor. Histologically, diagnosis of IMFT was a surprise.

IMFT or inflammatory pseudotumor is an unusual disease entity. The World Health Organization classification recognizes this disease, but very few cases in the temporal bone area have been published [3,4,15]. The common presenting symptoms of the disease are otalgia, otorrhea, hearing impairment, facial palsy, and vertigo [4,8]. Other symptoms include tinnitus, ear fullness, headache, retrobulbar pain, diplopia, facial pain, and visual dimness. However, an IMFT presenting with pulsatile tinnitus has not been reported previously (as researched by an Ovid Medline search using the medical subject headings ‘Pulsatile tinnitus/’ and ‘Inflammatory myofibroblastic tumor/Inflammatory pseudotumor’). To the best of our knowledge, this is the first such reported case.

A review of the literature suggests that the IMFT of the temporal bone has no distinctive characteristics, both clinically and radiologically. Thus, a diagnosis can only be made with histopathologic appearances and is confirmed by immunohistochemical studies [3]. The differential diagnoses include: Wegener’s granulomatosis, chronic osteomyelitis, sarcoidosis, cholesterol granuloma, cholesteatoma, histiocytosis X, eosinophilic granuloma, lymphoproliferative disorders, and other malignancies [10]. Treatment remains controversial. Surgical excision, steroid therapy, and radiation therapy have been employed, with the decision on treatment modality based on tumor location, size, and behavior [3]. As far as our patient is concerned, the diagnostic process is meandering. She had presented several times to different hospitals during a period of 10 years. Comprehensive medical evaluations including an earlier CT scan of the temporal bone had been carried out but failed to identity the underlying cause of her left pulsatile tinnitus, until a retrotympanic lesion was suspected and a second CT scan was performed in our hospital. This ultimately led to the exploration surgery and the final diagnosis of an IMFT. Fortunately, her pulsatile tinnitus was completely eliminated with radical excision of the lesion.

## Conclusions

IMFT of the temporal bone is an unusual but distinct disease entity. The most common presenting symptoms are otalgia, otorrhea, hearing loss, facial palsy, and vertigo. An IMFT can also present clinically with pulsatile tinnitus and masquerade as venous hum or vascular tumors of the middle ear, as in our case report. Therefore, an IMFT should be included in the differential diagnosis of pulsatile tinnitus, especially for those cases with a visible retrotympanic mass lesion.

## Consent

Written informed consent was obtained from the patient for publication of this case report and accompanying images. A copy of the written consent is available for review by the Editor-in-Chief of this journal.

## Competing interests

The authors declare that they have no competing interests.

## Authors’ contributions

GX analyzed and interpreted the patient data regarding the ear disease. XZ and TL were major contributors in writing the manuscript. ZC participated in the surgical treatment of the patient. ZZ performed the histological examination of the specimen. All authors read and approved the final manuscript.
